# Fauna used in popular medicine in Northeast Brazil

**DOI:** 10.1186/1746-4269-5-1

**Published:** 2009-01-07

**Authors:** Rômulo RN Alves

**Affiliations:** 1 Departamento de Biologia, Universidade Estadual da Paraíba, Av. das Baraúnas, 351/Campus Universitário, Bodocongó, 58109-753, Campina Grande-PB, Brazil

## Abstract

**Background:**

Animal-based remedies constitute an integral part of Brazilian Traditional Medicine. Due to its long history, zootherapy has in fact become an integral part of folk medicine both in rural and urban areas of the country. In this paper we summarize current knowledge on zootherapeutic practices in Northeast of Brazil, based on information compiled from ethnobiological scientific literature.

**Methods:**

In order to examine the diversity of animals used in traditional medicine in Northeast of Brazil, all available references or reports of folk remedies based on animals sources were examined. 34 sources were analyzed. Only taxa that could be identified to species level were included in assessment of medicinal animal species. Scientific names provided in publications were updated.

**Results:**

The review revealed that at least 250 animal species (178 vertebrates and 72 invertebrates) are used for medicinal purposes in Northeast of Brazil. The inventoried species comprise 10 taxonomic categories and belong to 141 Families. The groups with the greatest number of species were fishes (n = 58), mammals (n = 47) and reptiles (n = 37). The zootherapeutical products are used for the treatment of different illnesses. The most widely treated condition were asthma, rheumatism and sore throat, conditions, which had a wide variety of animals to treat them with. Many animals were used for the treatment of multiple ailments. Beyond the use for treating human diseases, zootherapeutical resources are also used in ethnoveterinary medicine

**Conclusion:**

The number of medicinal species catalogued was quite expressive and demonstrate the importance of zootherapy as alternative therapeutic in Northeast of Brazil. Although widely diffused throughout Brazil, zootherapeutic practices remain virtually unstudied. There is an urgent need to examine the ecological, cultural, social, and public health implications associated with fauna usage, including a full inventory of the animal species used for medicinal purposes and the socio-cultural context associated with their consumption.

## Background

Humans depend on biodiversity and the capacity of ecosystems to provide a multitude of goods and services that underpin a healthy human and natural environment. Biodiversity is essential for human health, for example, in the provision of the raw materials for medicines. Indeed, some 20,000 species are used in traditional medicine, which forms the basis of primary health care for about 80 percent of the 3 billion people in developing countries. More than half of the world's modern drugs are derived from biological resources, which supports the traditional and modern pharmaceutical sectors [1,2].

Plants and animals have been used as medicinal sources since ancient times [2-5], and even today animal and plant-based pharmacopeias continue to play an essential role in world health care [6]. Although plants and plant-derived materials make up the majority of ingredients used in most traditional medical systems globally, whole animals, animal parts, and animal-derived products (e.g., urine, fat, etc.) also constitute important elements of the materia medica. Indeed, zootherapy, the use of animal products in healing, is an ancient and widespread practice across most cultures [3,5,7,8].

Animals have been broadly used since ancient times in Brazilian traditional medicine [9], and have played a significant role in healing practices [9-11]. Expressions of traditional medicine in the country, particularly of zootherapy, represent an interaction of native, African and European elements, since the beginning of colonization [9], resulting in a rich ethnomedicine used by people belonging to different social classes in Brazil [12]. Nevertheless, the use of animal species as remedies, although representing an important component of traditional medicine (sometimes in association with plant species), has been much less studied than medicinal plants in the country [11,13-15].

Little attention has been paid to the cultural, medical, economic, or ecological significance of zootherapeutic practices, even though the federal government's National Policy of Pharmaceuticals (Política Nacional de Medicamentos, Portaria no. 3916/98) specifies that "the support to research aiming to use the therapeutic potential of the national flora and fauna, with emphasis on certification of their medical properties, should be continued and expanded" [11]. Nevertheless, since the 1980s various publications have shown the importance of zootherapy for traditional communities from distinct socio-cultural-environmental landscapes in Brazil. Most of the available information on the subject is concentrated in the Northeast of the country [13].

In addition, the edibility of these medicinal resources must be analyzed because there must be complex interactions between diet and the medicinal use. A number of food animals are also used as remedies [11,13-17]. Yet, our knowledge about the practice of food medicine is limited, particularly with regards to the traditional consumption of animal food-medicines [18]. Although often regarded as supplementary to local peoples' diet, wild food and medicine are essential in times of crisis and play an important nutritional role. The neglect of traditional food and medicines may seriously deteriorate the health and well being of traditional peoples [19,20]. Furthermore, nature-based traditional food and medicine are generally viewed as interchangeable, diet being highly regarded as the primary basis for sustaining and/or restoring health and well-being. Consequently, foods are considered and often times chosen for their distinctive medicinal or healing values.

Given Brazil's significant cultural and biological diversity, the country can be used as a useful case study to increase our knowledge of faunistic resources used as medicines, and to draw attention to the need to protect traditional knowledge and biodiversity [13]. In that context, the aim of this paper is summarize current knowledge on zootherapeutic practices in of Northeast of Brazil, based on information compiled from ethnobiological scientific literature, aiming to establish a regional data base. Contributions is expected in order to increase our knowledge concerning the faunistic resources used in the traditional medicine in the country, alerting for the need of protecting the biodiversity and the traditional knowledge and still emphasize the importance of a therapeutic modality that although widely disseminated at the country, is getting little attention from the scientific community.

## Methods

### Study area

**Figure 1:**
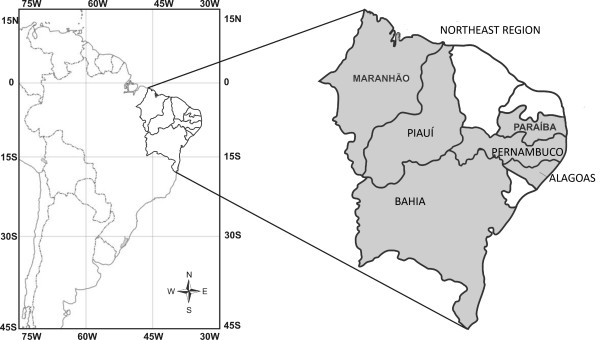
**Map of Northeast Brazil, showed states where researches on zootherapy were carried**.

The total area of the Brazilian Northeast is 1,561,177.8 km^2^, which extends from 02°54 to 17°21S and from 35° to 46°30W and includes nine States: Maranhão, Piauí, Ceará, Rio Grande do Norte, Paraíba, Pernambuco, Alagoas, Sergipe and Bahia (Figure. 1) [21]. This region is home to around 51 million people, representing 28.9% of the total population of Brazil, most of whom live in the urban area. The inhabitants of Northeast Brazil exhibit a high degree of race mixing. According to the 2006 census of Brazilian Institute for Geography and Statistics (IBGE), people of multiracial (European, Amerindian and African) background make up 62.5% of the population, while those of total or predominantly Black ancestry account for 7.8%. This region was not heavily affected by the wave of European immigration that took place in Southern Brazil during the 19th century – the Northeast was (and still is) the poorest part of Brazil, and therefore there was little incentive for new immigrants to stay [22].

As a result of the huge land mass involved, the diverse physio-geography of the region, as well as the conjunction of two major weather systems, provided by the NE and SE trade winds, rainfall patterns in Northeast Brazil are typically diverse and instable. The precipitation within the region varies from being extremely wet, with an annual rainfall of up to around 2,000 mm along the coast, to only 300–500 mm in the semi-arid zone, where the rainfall is usually restricted to a few months during the year. The availability of water determines the type and abundance of vegetation and fauna that exists in the region, as therefore in turn the patterns of human exploitation of natural resources [23].

The predominant vegetation type in this region is composed of several forms of caatinga biome. The structure of these forests can vary considerably from forests composed of mostly spiny trees, 6 to 10 m tall, often with a ground-layer of small deciduous shrubs and annual herbs, predominantly Leguminosae, to deciduous woodlands of lower stature, with a high proportion of shrubs and subshrubs and the presence of many cacti, bromeliads and Euphorbiaceae [24].

The Caatinga has been described as harboring relatively few species and having low numbers of endemic species [25-27]. Some recent studies, however, have challenged this and demonstrated the importance of the region for the conservation of a significant component of Brazilian biodiversity [28]. Inventories and assessments have, to date, recorded 932 vascular plant species [29], 187 bees [30], 240 fishes [31], 167 reptiles and amphibians [32], 62 families and 510 species of birds [33], and 148 mammal species [34]. Levels of endemism vary from about 7% for mammals [34,35] to 3% in birds [33] and 57% in fishes [31]. The Northeast region as a whole holds more types of vegetation than any other region in Brazil. In addition to the Caatinga biome, there are the Atlantic Rainforests, seasonal forests and inland mountain forests, *restinga *and shore dunes, mangroves, cerrados (savannah-like vegetation) and 'campos rupestres', all of which exhibit rich animal and plant biodiversity.

### Data Collection

In order to examine the diversity of animals used in traditional medicine in Northeast of Brazil, all available references or reports of folk remedies based on animals sources were examined [9-15,36-62]. 34 ethnobiological sources documenting the medicinal use of animals were analyzed. Only taxa that could be identified to species level were included in the data base. Scientific names were updated in accordance with the Integrated Taxonomic Information System's "Catalogue of Life: 2008 Annual Checklist" [63]. The conservation status of the animal species follows IUCN [64], Convention on International Trade in Endangered Species of Wild Fauna and Flora – CITES [65], Brazil's official list of endangered species [66], and the national list of species of aquatic invertebrates and fishes endangered, overexploited, or threatened by exploitation [67].

The reputed therapeutic effects and ailments treated were grouped into 20 categories (Table 1) based on the classification used by the Centro Brasileiro de Classificacão de Doenças (Brazilian Center for the Classification of Diseases) [68], following Alves and Rosa [13-15].

**Table 1:** Categories of diseases treated with zootherapeutic remedies in Northeast Brazil, according to the Brazilian Centre of Diseases Classification and the number of species used per category.

**Categories of diseases**	**Number of medicinal animals**
Some infections and parasitic diseases	58
Respiratory system	132
Digestive system	38
Undefined illnesses	71
External causes of morbidity and mortality	45
Osteomuscular system and conjunctive tissue	71
Injuries, poisoning and other consequences of external causes	77
Urinogenital system	19
Circulatory system	64
Skin and subcutaneous tissue	44
Nervous system	27
Neoplasias (tumours)	12
Ear (middle and inner ear) and mastoid apophysis	32
Blood and haematopoeitic organs, and some disorders of the immune system	4
Pregnancy, parturition, and puerperium	7
Symptoms, signs, and abnormal findings from medical and laboratorial examination, not categorized in other part or section	9
Mental and behavioral perturbations	12
Diseases of the endocrine glands, metabolism and nutrition	3
Ophthalmological diseases	13
Congenital malformations, deformities and cromossomic abnormalities	2

## Results and discussion

**Figure 2:**
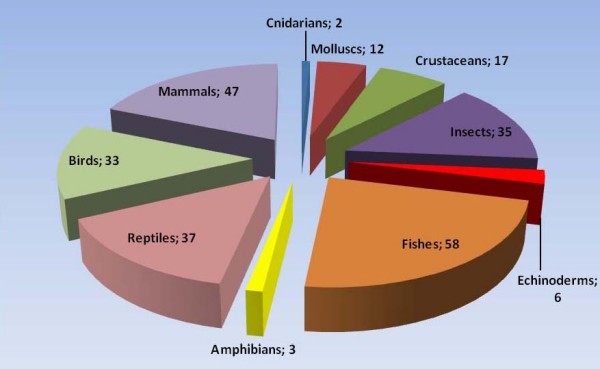
**Number of animal species used as remedies per taxonomic category in Northeast Brazil**.

**Figure 3:**
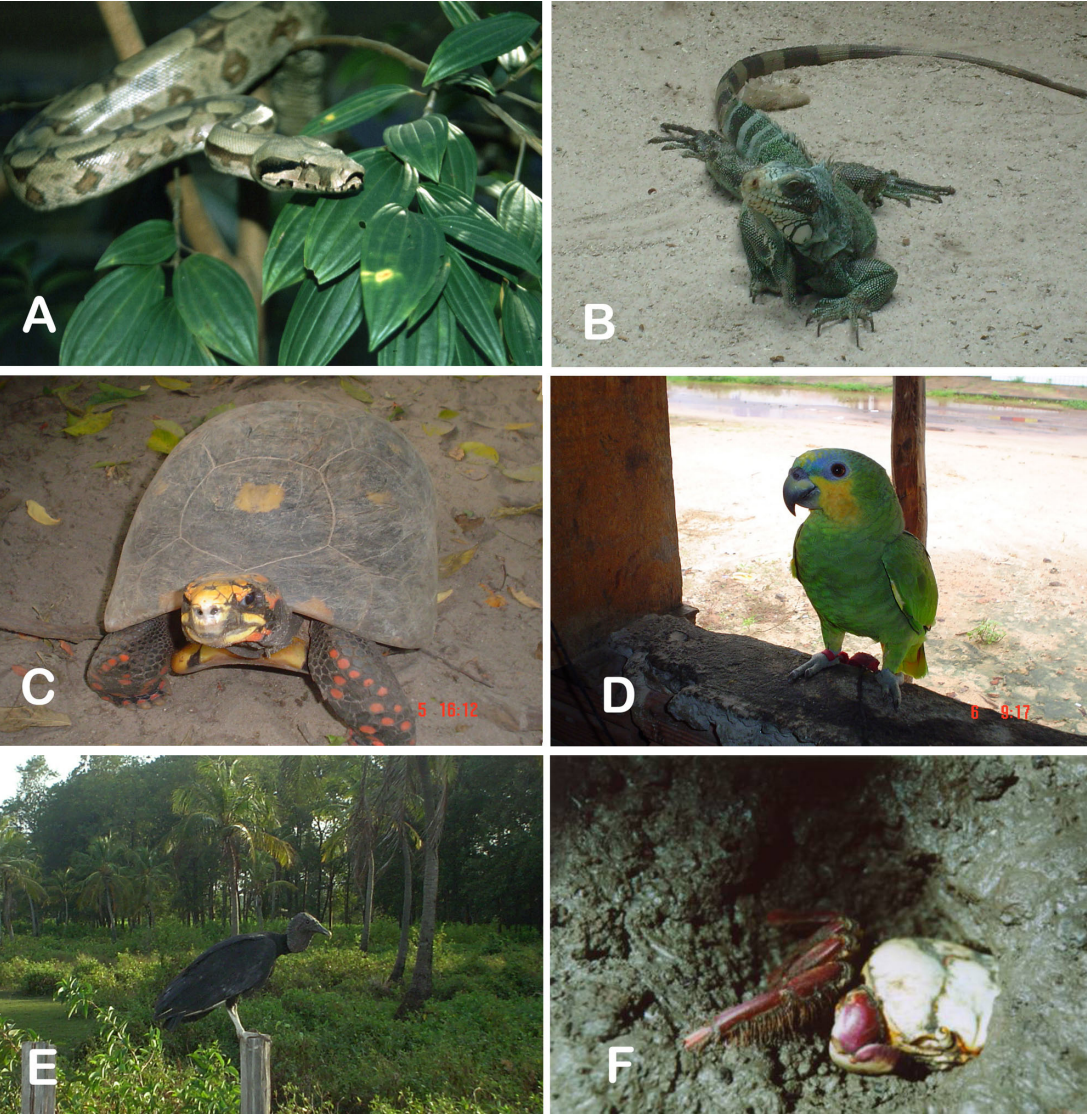
**Examples of animals used as medicine in Northeast Brazil**. A: *Boa constrictor *(Photo: Gentil A. Pereira-Filho), B: *Iguana iguana*, C: *Chelonoidis carbonaria*, D: *Amazona aestiva*, E: *Coragyps atratus *and F: *Ucides cordatus *(Photos B,C,D,E,F: Rômulo R.N. Alves).

The review revealed that at least 250 animal species (178 vertebrates and 72 invertebrates) are used for medicinal purposes in Northeast of Brazil. The inventoried species comprise 10 taxonomic categories and belong to 141 Families (Additional file 1). The groups with the greatest number of species were fishes (n = 58), mammals (n = 47) and reptiles (n = 37) (Figure. 2). These results are in line with previous studies carried out around the world, as numerous workers have pointed out that vertebrates are the animals most frequently used in folk medicine [69-75]. Examples of animals used as medicine is showed in Figure 3.

The number of medicinal species catalogued was quite expressive and demonstrate the importance of zootherapy as alternative therapeutic in Northeast of Brazil. Ethnobiological studies encompassing information on the medicinal use of biological resources cover 06 states: Paraíba, Piauí, Pernambuco, Alagoas, Maranhão and Bahia, the latter being state with the highest number of studies (Figure. 1). No published accounts were found for the states of Ceará, Sergipe and Rio Grande do Norte. Due to the lack of studies in some states of Northeast Brazil, and to the fact that only taxa that could be identified to the species level were included in the review, is expected the number of medicinal animals to be greater than the 250 species compiled.

Of the 250 medicinal animal species which have been recorded, 175 (70%) were also used as food. The high number of animals used both as food and medicine is not surprising given the important role played by wildlife as a source of protein in different parts of the world. In at least 62 countries worldwide, wildlife (including fish) provides significant proteins, calories, and essential fats to rural communities [18,76-82]. The extensive use of foods as medicinal remedies reported in our study is in line with recent field investigations around the world [16-18]. The degree of overlap between medicinal and nutritional uses of wild animals observed in our study was high, and left no doubt about the importance of wild animals in human diets and healing activities.

Also, this work showed that exists a larger knowledge on medicinal animals in predominantly rural areas, nevertheless, also drew attention to the zootherapeuticals knowledge of the urban poor in cities across the Northeast region [10-12,15,40,41,48,55,60,81,82]. The notable use and commercialization of medicinal animals to alleviate and cure health problems and ailments in the cities of Brazil reveals the resilience of that therapeutic alternative, in spite of the influence of the western medicine. In urban areas, the people brought from their villages to the cities much valuable knowledge on animals-based remedies that is rarely studied. The use of similar resources as medicines in more remote and urban areas suggest that zootherapeutic practices may function as a social conduit which, in conjunction with other factors, helps to maintain the connections between rural and peasant people living in cities and their own traditional culture and values. More specifically, it indicates the potential for exchange of materials and information on illnesses and treatments between more remote and urban communities [15].

**Figure 4:**
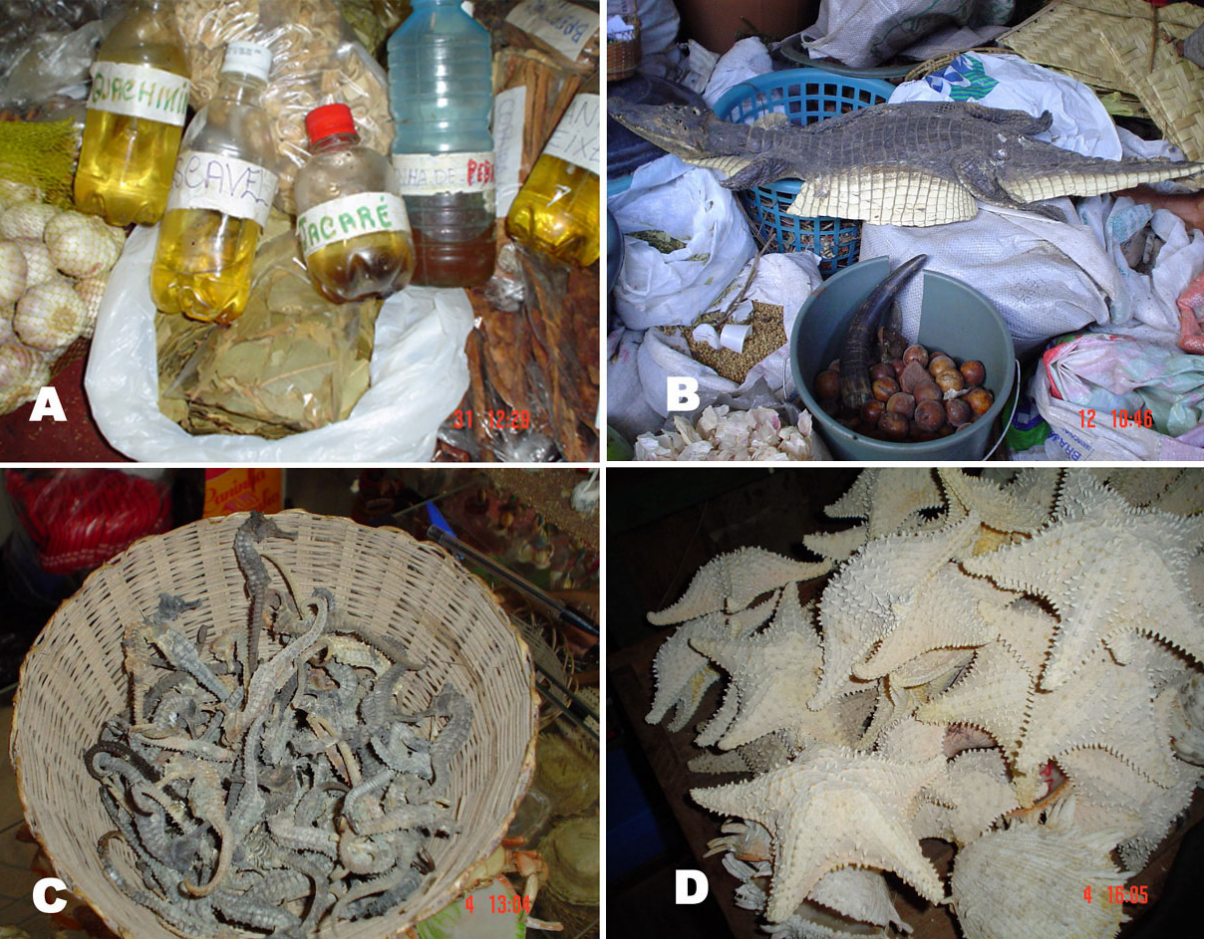
**Examples of animal products used as remedies in Northeast Brazil**. A: Fats of mammals and reptiles, B: Alligator leather (*Paleosuchus palpebrosus*), C: Dried Seahorses (*Hippocampus reidi*) and D: Dried starfish (*Oreaster reticulatus*).

Medicinal animals recorded can be used whole or in parts such as fat, flesh, bone, bone marrow, cartilage, skin, tail, feather, liver, bile ("fel"), milk, rattle (from rattlesnakes), spine, shell, honey, wax, scale, rostral expansion, otolith, penis, carapace, blood, gizzard, beak, cocoon, teeth, tongue, egg, egg shells, tibia, secretions, head, heart, urine, foot, leg, nest, guts, pollen, ear, spawn, nail, horn, sucking dish and eye. Examples of zootherapeutic products used as remedies in Northeast Brazil are showed in Figure 4. Similarly, the most of zootherapeutic resources used in Northeast of Brazil have been recorded in studies previous in others regions of country, and of the world [3,69-75,81-88].

Beyond the use for treating human diseases, zootherapeutical resources are also used in ethnoveterinary medicine. Barboza et al. [59] recorded the utilization of animals (zootherapeutics) as sources of medicines in folk veterinary medicine (ethnoveterinary) in semiarid northeast region and verified that 15 animals are used in the prevention or cure of animals' illnesses in that region.

Distinct preparation and administration manners of the zootherapic resources are reported in the works, but in general, hard parts, such as teeth, nails, shells, rattles from snakes, fish scales, bone, and cartilage, generally are dried in the sun, grated, and crushed to powder, and then administered as tea or taken during meals. Fat, body secretions, and oil are either ingested or used as an ointment. Some animals are utilized in combination with plants and/or other animal species, constituting the ingredients of what the interviewees call "garrafadas" a concoction defined by Camargo [89] and Ngokwey [90], as a therapeutic drink composed of various plants soaked in *cacha, ca *(Brazilian sugar cane liquor) or white wine and contained in a bottle (*garrafa *in portuguese, hence the name *garrafada*). The uses of medicinal plants and animals overlap in many cases [11,13-15,62], as might be expected, as phytotherapy and zootherapy are well known and widely used therapeutic alternatives in contemporary societies [5]. Considering the fact that the use of medicinal animals and plants is quite common in most areas of Northeast Brazil [11,91,92], various overlapping usages might well be expected among traditional remedies [11,13,41].

The zootherapeutic resources recorded were used to treat different diseases. The most widely treated condition were asthma, rheumatism and sore throat, conditions, which had a wide variety of animals to treat them with. Many animals were used for the treatment of multiple ailments. The highest numbers of animal species (132, 52.8%) have been reported for the treatment of Respiratory system related problems. Injuries, poisoning and other consequences of external causes are treated with 77 species (30.8%). 71 (28.4%) animal species are reported in uses in Undefined illnesses category (that includes all citations for diseases with unspecific symptoms). Problems of osteomuscular system and conjunctive tissue are reported to be treated with 71 (28.8%) species. Circulatory system related problems are treated with 64 species (25.6%) (Table 1).

Sanitary conditions of the zootherapeutics products generally were poor with obvious contamination risks to these products [11,13,15,93]. These observations point to the need for sanitary measurements to be taken with medicinal animal products and the importance of including considerations about zootherapy into public health programs. Although the need for implementation of sanitary measures to the trade of animal or their parts for medicinal purposes is evident, adoption of regulatory measures faces considerable challenges, among them ensuring adequate participation of all stakeholders involved, monitoring of the activity, and combating illegal, unreported and unregulated trade [5]. Additionally, chemical and pharmacological studies are necessary in order to clarify the eventual therapeutic usefulness of this class of biological remedies [17]. The possibility of using various remedies for the same ailment is popularly valued [89], as it renders an adaptation to the availability/accessibility of animals possible [11].

The economic and geographic accessibility of medicinal animals, perceived efficacy and sociocultural factors were main reasons for popularity of zootherapy [11,13-15]. Because Brazil is highly heterogeneous socially and profoundly unequal in distribution of income, socioeconomic aspects play a role in the perseverance of zootherapeutic practices [11]. For the majority of the population, access to hospital care is available within the public sector, but the organization of the health-care system reflects the schisms within Brazilian society: high-technology private care is available to the rich, but only inadequate public care is available to the poor [94,95], which makes the use of available, affordable animal and plant remedies an important alternative.

The high number of species registered evidenciates that the animals are therapeutic resources culturally important. Nevertheless, the lack of zootherapeutic studies in Brazil (and in the world in general) has contributed to an underestimation of the importance of zootherapeutic resources in this country. Alves and Rosa [15], suggest that one of the factors that certainly contribute to the information scarcity on the subject is the semi-clandestine or clandestine nature of the trade and use of medicinal animals, generally result in usuaries and traders being more resistant to provide information. The most of medicinal animals are wild and protected by law. Nevertheless, although Brazilian legislation forbids commercial use of wild fauna (Article 1 Law 5,197 January 3, 1967 and Article 29 of Law 9,605 February 12, 1998), medicinal products and derivatives made from animals are commonly traded in many Brazilian cities [10-12,15,41,41,48,55,60-62].

Most of the species used (*n *= 230; 92%) are wild caught. In most cases remedies were prepared from dead specimens. Many of the medicinal animals are of conservation concern. Many of the recorded species (52 out of 250) are on either the IUCN Red List of Threatened Species, [64] CITES list (Convention on International Trade in Endangered Species of Wild Fauna and Flora [65], Brazil's official list of endangered species [66], or the National List of species of aquatic invertebrates and fishes endangered, overexploited or threatened by exploitation [67]. These results demonstrate the need to assess the implications of the use and trade of animal used in traditional medicines on their wild populations.

As pointed out by Alves et al [11], there is a need to increase our understanding of the biology and ecology of species commonly used as remedies to better assess the impacts of harvesting them (for medicinal or other purposes) on their wild populations. Medicinal species whose conservation status is in question should receive urgent attention, and aspects such as habitat loss and alteration should be discussed in connection with present and future medicinal uses. As Anyinam [4] remarked, environmental degradation affects users of traditional medicine both by limiting their access to the resources traditionally used and by diminishing the knowledge base in their community upon which traditional medicine is constructed. Studies on traditional uses of faunistic resources should be carried out with other links to conservation biology, public health policies, sustainable management of natural resources and biological prospection is of great importance [96].

## Conclusion

A total of 250 animals are used for medicinal purposes in the Northeast of Brazil, evidencing that the zootherapy represents a traditional practice in the region. The high number of animal species registered reveals the cultural importance of that practice as therapeutic alternative, being occasionally used in association with medicinal plants. In a country like Brazil, where the majority of the population has no access modern allopathic medicines, local medicinal animals and plant knowledge systems is of significance. The population uses traditional medicine due to the relatively low cost of traditional medicine and difficult access to modern health facilities. Nevertheless, the interest in and intrinsic value of zootherapy not be only be attributed to the lack of access to modern medicinal services. Even in cities where modern health services are more accessible and specialized; many people continue to go to traditional healers showing the cultural acceptability of such practices. Besides the biological aspects, the economical and sociocultural factors influence the relationship of the local gathered people and the zootherapic resources usage. The need of new studies is evidenced which approach the medicinal fauna of Brazil, seeking for a better understanding of this therapy form, not only in its ecological aspects, but also cultural and pharmacological.

## Competing interests

The author declares that they have no competing interests.

## Supplementary Material

Additional file 1 **Medicinal animals and its respective uses in popular medicine, Northeast of Brazil.** The data provided a list of medicinal animals and its respective uses in popular medicine in the Northeast of Brazil. Click here for file
