# Supplementary material for: Fauna used in popular medicine in Northeast Brazil
Source: J Ethnobiol Ethnomed. 2009 Jan 7;5:1. doi: 10.1186/1746-4269-5-1 (PMC2628872; doi:10.1186/1746-4269-5-1)
Supplement: Additional file 1 — Medicinal animals and its respective uses in popular medicine, Northeast of Brazil. The data provided a list of medicinal animals and its respective uses in popular medicine in the Northeast of Brazil. [file 1746-4269-5-1-S1.pdf]

Additional file 1/Table 1 - Animal taxa recorded as having medicinal properties

Family / Species / local name	Conditions to which remedies are prescribed	References
CNIDARIANS		
Mussidae		
Mussismilia harttii (Verril, 1868)[NE/II] – “Coral branco”	Vaginal discharge, diarrhoea	9,
Physaliidae		
Physalia physalia (Linnaeus, 1758) - Portuguese-man-of-war, “caravela”	Asthma	11,13,14, 15
MOLLUSCS		
Ampullariidae		
Pomacea lineata (Spix, 1827) - Snail, “aruá”, “caramujo”	Asthma, sprains, boils, ulcer	11, 13,14, 36, 37, 46, 57
Cassidae		
Cassis tuberosa (Linnaeus, 1758) - “Concha”	Asthma	56
Littorinidae		
Littorina angulifera (Lamarck, 1822) - Periwinkle snail, “mela-pau”	Chesty cough, shortness of breath	11,13,14
Lucinidae		
Lucina pectinata (Gmelin, 1791) – “Lambreta”, “marisco redondo”	Sexual impotence	46
Melongenidae		
Pugilina morio (Linnaeus, 1758) – “Rochelo”	Sexual impotence	46
Mytilidae		
Mytella charruana (Orbigny, 1842) – Mussel, “marisco”, “sururu”	Ophthalmological problems	56,
Mytella guyanensis Lamarck (1819) - Mussel, “sururu”	Weakness	11,13,14,15
Ostreidae		
Crassostrea rhizophorae (Guilding, 1828) - Mangrove oyster, “ostra do mangue”	Osteoporosis, pneumonia, stomach ache, cancer, flu, weakness, pain relief in injuries caused by the dorsal fin spine of a species of	11,13,14, 15

	catfish, anaemia, tuberculosis	
Strombidae		
Strombus puginis Linnaeus, 1758 – "Taquari"	Sexual impotence	46
Teredinidae		
Teredo pedicellata Quatrefages, 1849	Tuberculosis	56
Vasidae		
Turbinella laevigata (Anton, 1839) – "Tapu"	Sexual impotence	46
Veneridae		
Anomalocardia brasiliana (Gmelin, 1791) – Clam, "marisco"	Asthma, flu, stomach ache	11,13,14
CRUSTACEANS		
Calappidae		
Calappa ocellata Holthuis, 1958 – "guajá"	Asthma, osteoporosis	10
Gecarcinidae		
Cardisoma guanhumi Latreille, 1825[NE/IN] – "Goiamum", "gaiamun"	Asthma, bronchitis, wounds, boils	52, 46
Grapsidae		
Goniopsis cruentata (Latreille, 1802) - mangrove root crab, "aratu do mangue", "caranguejo-aratu"	Epilepsy, venereal disease	11,13,14, 56
Plagusia depressa (Fabricius, 1775) - "caranguejo-aratu"	Epilepsy	56
Hippidae		
Emerita portoricensis Schmitt, 1935	Earache	56
Ocypodidae		
Ocypode quadrata (JC Fabricius, 1787) – Ghost crab, "maria farinha", "grauçá"	Asthma, haemorrhage in women, flu, asthma, to alleviate the symptoms of intoxication with poison of 'niquim' (Pisces, Batrachoididae)	11,13,14, 46, 50, 52
Ucides cordatus (Linnaeus, 1763)[DD/IN] - Land crab, "caranguejo-ucá"	Hemorrhage in women, incontinence, osteoporosis, cough, asthma, tuberculosis, womb disorders, arthrosis, bronchitis	11,13,14, 46, 50, 52
Uca maracoani (Latreille, 1802) - Fiddler	Asthma, whooping cough	11,13,14

crab, “maracoani”		
Palaemonidae		
Macrobranchium carcinus (Linnaeus, 1758) – “Pitu”	Amnésia	56
Macrobrachium acanthurus (Wiegmann, 1836) - Cinnamon river shrimp, “camarão de água doce”	Irritation when milk teeth are erupting	11,13,14
Macrobrachium borellii (Nobili, 1896) - Freshwater shrimp, “camarão de água doce”	Irritation when milk teeth are erupting	11,13,14
Penaeidae		
Xiphopenaeus schmitti (Burkenroad, 1936), Southern white shrimp, “camarão branco”	Irritation when milk teeth are erupting, skin spots	13,14, 11,
Xiphopenaeus kroyeri (Heller, 1862) - Atlantic seabob, “camarão branco”	Irritation when milk teeth are erupting, skin spots	11,13,14
Pseudosquillidae		
Cloridopsis dúbia (H. M. Edwards, 1837) – “Barata-do-mar”	Asthma	12,46, 48
Sesarmidae		
Aratus pisoni (H. Milne Edwards, 1837) - Mangrove crab, “aratu preto”, “caranguejo-aratu”	Epilepsy, to alleviate the symptoms of intoxication with poison of *Colomesus psittacus* (a species of pufferfish)	11,13,14, 56
Trichodactylidae		
Trichodactylus fluviatilis (Latreille, 1828) - caranguejo de água doce, freshwatercrab	Asthma	54
Armadilloidea		
Armadillidium vulgare (Latreille, 1804) - tatuzinho, pillbug	Asthma	61
INSECTS		
Apidae		
Apis mellifera (Linnaeus, 1758) - Africanised honey bee, “abelha italiana”	Cough, flu, rheumatism, tuberculosis, bronchitis, hoarseness, ulcer, diabetes, verminosis, headache, giddiness, backache, wounds, burns, mumps, varicose veins, arthrosis, cellulitis, amoebiasis, sore throat,	11,13,14, 15, 36, 37, 46, 47, 49, 51, 53, 57, 58, 60, 61,62

	ashtma, anaemia	
Cephalotrigona capitata (Smith, 1854) - "Abelha papa-terra"	Snake bite (antidote)	46, 47
Frieseomelitta silvestrii (Friese, 1902)	Flu	47
Frieseomelitta varia (Lepeletier, 1836) – Bee, Abelha moça branca	Flu	60
Melipona compressipes (Fabricius, 1804) - Stingless bee, "tiúba"	Asthma, cough	11,13,14, 15
Melipona mandacaia Smith, 1863 – Bee	Wounds	45
Melipona quadrifasciata Lepeletier, 1836	Snake bite	47,
Melipona scutellaris (Latreille, 1811) - Stingless bee, "urucu"	Headache, migraine, stroke, verminosis, stomach ache, tuberculosis, haemorrhage after delivery, cataracts, mycosis in the mouth, flu, cancer, asthma, bronchitis, intestinal disorders, cough, sexual impotence, mycosis in the mouth; ophthalmological problems, weakness, thrombosis, migraine, amoebiasis, snake bite (antidote), rabies	11,13,14,15, 36, 37, 41, 44, 45, 46, 47, 49, 53, 57,60
Melipona subnitida (Ducke, 1910) - "Jandaíra"	Flu, sore throat	11,13,14, 15,51
Partamona cupira - Stingless bee, "cupira"	Sore throat, swelling, headache, thrombosis, stroke	46, 47, 60,62
Plebeia cf. *emerina* Friese, 1900 – "Mosquito remela"	Mycosis in the mouth area	47,
Tetragonisca angustula Latreille, 1811 – "Abelha-jataí"	Cataracts, sinusitis, cough, flu, ophthalmological problems, sore throat	11,13,14,15, 45,60
Trigona mosquito Lutz, 1931 - Stingless bee	Cough	53
Trigona spinipes (Fabricius, 1793) - Stingless bee, "arapuá"	Asthma, cough, flu, bronchits, acne, diabetes, strokes, thrombosis, migraine, itching, sore throat, giddiness, weakness, scabies, fleas in dogs, nasal congestion, to induce abortion, whooping cough, irritation when milk teeth are erupting, earache, epilepsy, shortness of breath, late menstruation	12, 36, 37, 44, 45, 46, 47, 48, 49, 53, 57,58,60
Blattidae		
Periplaneta americana (Linnaeus, 1758) –	Heartburn, asthma, stomach ache, intestinal	11,13,14, 44, 45, 46, 47, 53,60,61

American cockroach, “barata”	colic, earache, alchoolism, epilepsy, vomit, boil, haemorrhage, bronchits, diarrhea, gonorrhea, panaris, cancer, stroke, burns, menstrual cramps, wounds, to suck a splinter out of skin or flesh, detoxification (alcohol abuse)	
Chrysomelidae		
Coraliomela brunnea Thumberg, 1821 – “barata-de-coqueiro”	Epilepsy	11, 15
Pachymerus cf. *nucleorum* – “bicho-do-ouricuri”	Earache, stroke, swelling, wounds, seborrheic dermatitis, inflammation, thrombosis	45, 46, 53
Curculionidae		
Rhynchophorus palmarum Linnaeus, 1758 – “bicudo”, “broca-do-olho-coqueiro”	Fever, headache, boils	9
Rhinostomus barbirostris Fabricius, 1775 – “broca-do-estipe”	Fever, headache, boils	9
Formicidae		
Atta cephalotes (Linnaeus, 1758), Leaf-cutter ant, “tanajura”	Sore throat	11,13,14. 62
Atta serdens (Linnaeus, 1758) - Leaf-cutting, “saúva-ataí”	Stomach ache, heart diseases, chest palpations	53
Dinoponera quadriceps (Santschi, 1921) - “Trinca cunhão”	Asthma	11,13,14,15
Gryllidae		
Acheta domesticus (Linnaeus, 1758) - “Grilo”	Scabies, asthma, eczema, lithiasis, earache, oliguresis, rheumatism, urine retention, children that urinate in bed and speak with lateness, incontinence urinary, ophthalmological problems	53
Meloidae		
Palembus dermestoides (Fairmaire, 1893) - Peanut beeatle, “besouro-do-amendoim”	Sexual impotence, ophthalmological problems, rheumatism, weakness	46, 53
Muscidae		
Musca domestica (Linnaeus, 1758) - “Mosca comum”	Boil, baldness, eyesore, external sebaceus lamps, stye, spots in the face, ophthalmological problems, dermatosis, cysties	44, 46, 53,60,61

Psychidae		
Eurycotis manni Rehn, 1916 – “Carocha”	Headache	45
Oiketicus kirbyi Guilding, 1827 - Case moth, “caixinha”, “chocalho de cobra”, “baia”, “lagarta-encantada”	Asthma, earache, haemorrhage after delivery	53
Termitidae		
Microcerotermes exignus (Hagen, 1858), - Térmite, “cupim”	Asthma, bronchitis, flu, whopping cough	53
Nasutitermes macrocephalus (Silvestri, 1903) - Termite, “cupim”	catarrh	60, 62
Vespidae		
Apoica pallens (Oliv., 1791) - Paper wasp, “marimbondo-chapéu”, “eixu-chapéu”	Thrombosis, ashtma, giddiness, nasal haemorrhage, haemorrhage after delivery, stroke, disorders after parturition (to accelerate recovery after parturition), ophthalmological problems, mumps, late menstruation	45, 46, 47, 53
Brachygastra lecheguana (Latreille, 1824) – “Inxu-verdadeiro”, “marimbondo-de-pote”	Cough, asthma	53
Polistes canadensis (Linnaeus, 1758) – “Marimbondo-santa-maria”	Cough, whooping cough	52, 46
Polybia sericea (Olivier, 1791) - “Tarantantã”	Thrombosis	46, 47
Protopolybia exigua (Saussure, 1854) – “Inxu-cachorro”	Evil eye, tobaccoism, ophthalmological problems	46, 47
Synoeca surinama (Linnaeus, 1767) – Paper wasp, “marimbondo-tatu”	Asthma, shortness of breath	53
ECHINODERMS		
Echinasteridae		
Echinaster brasiliensis Müller & Troschel, 1842 – “Estrela-do-mar”	Asthma	10, 12, 46
Echinaster echinophorus Lamarck, 1816 – “Estrela-do-mar”	Asthma	46
Echinometridae		
Echinometra lucunter (Linnaeus, 1758) – “Pinaúna”, “ouriço-do-mar”	Asthma	10, 46, 50, 52
Luidiidae		

Luidia senegalensis Lamarck, 1916 - "Estrela-do-mar"	Asthma, cough, metrorrhagia	11,13,14,15, 46, 50, 52,61
Mellitidae		
Mellita sexiesperforata (Leske, 1778) – "Estrela-da-costa", "bolacha-da-praia"	Asthma, cough	56
Oreasteridae		
Oreaster reticulatus (Linnaeus, 1758) - Starfish, "estrela-do-mar"	Asthma	11,13,14, 15,60
FISHES		
Auchenipteridae		
Trachelyopterus galeatus (Linnaeus, 1766) - "Cumbá"	Umbilical hernia, asthma, sexual impotence	36, 37, 46, 49, 57,58,61
Anostomidae		
Schizodon knerii (Steindachner, 1875) - "Piau-branco"	Leucoma, edema	57
Ariidae		
Bagre bagre (Linnaeus, 1766) - Coco sea catfish, "bagre-fidalgo"	Injuries caused by itself	46, 50, 52
Genidens barbus (Lacepède, 1803) – White sea catfish, "bagre-do-mangue"	Pain relief caused in injuries by the species' sting	46, 52
Genidens genidens (Cuvier, 1829) – catfish, "bagre"	Injuries caused by itself	42
Sciadeichthys luniscutis (Valenciennes, 1837) – "bagre-urutu"	Pain relief caused in injuries by the species' sting	46, 52
Aspredinidae		
Aspredo aspredo (Linnaeus, 1758) – Banjo catfish, "banjo", "viola"	Asthma	11,13,14
Aspredinichthys tibicen (Valenciennes, 1840) - Tenbarbed banjo, "viola"	Asthma	11,13,14
Balistidae		
Balistes vetula (Linnaeus, 1758)[VU] - Queen triggerfish, "cangulo", "capado", "peroá"	Stroke, asthma, thrombosis, earache, pain relief caused in injuries by the species' sting, haemorrhage, ascites, schistosomiasis, appendicitis, menstrual cramps, gastritis	11,13,14, 15, 46, 50, 52
Batrachoididae		
Thalassophryne nattereri (Steindachner, 1876) - Venomous toadfish, "niquim"	Pain relief caused in injuries by the species' sting	11,13,14, 46, 50, 52

Callichthyidae		
Callichthys callichthys (Linnaeus, 1758) – “cascarudo”, “caboge”	Asthma, umbilical hernia	46, 50, 52, 57
Carcharhinidae		
Carcharhinus limbatus (Müller & Henle, 1839) – Blackfin shark, “sucuri preto”	Osteoporosis	11,13,14
Carcharhinus porosus (Ranzani, 1840) - Smalltail shark, “junteiro”, “cação-gaia-preta”	Asthma, rheumatism, wounds, inflammations, osteoporosis, anaemia	11,13,14, 52
Galeocerdo cuvier (Péron & Lesueur, 1822) - Tiger shark, “jaguara”	Osteoporosis	11,13,14
Rhizoprionodon lalandii (Müller & Henle, 1839) - Brazilian sharpnose shark, “cação”	Rheumatism	11,13,14
Rhizoprionodon porosus (Poey, 1861) – Sharpnose shark, “cação”	Rheumatism	11,13,14
Sphyrna lewini (Griffith & Smith, 1834) [IN] - Scalloped hammerhead, “peixe-martelo”, “cação-panã”, “cação-chapéu”	Asthma, wounds, rheumatism, inflammation	46, 50, 52
Centropomidae		
Centropomus parallelus Poey, 1860	Nephritis	9
Centropomus undecimalis (Bloch, 1792) - Common snook, “rubalão”	Edema in the legs	46, 50, 52
Characidae		
Astyanax bimaculatus (Linnaeus, 1758) - Twospot astyanax, “piaba-mirim”, “machadinha”, “piaba chata”	Alcoholism, leishmaniosis, skin burns, wounds, rheumatism	46, 52
Brycon nattereri Günther, 1864 – “pirapitinga”, “matrinchã”	Flu	43
Clupeidae		
Opisthonema oglinum (Lesueur, 1818) - Atlantic thread herring, “sardinha”	Alcoholism	11,13,14
Dasyatidae		
Dasyatis guttata (Bloch & Schneider, 1801) - Longnose stingray, “raia branca”	Asthma, pain relief caused in injuries by the species’ sting, burns	11,13,14
Dasyatis marianae (Gomes, Rosa & Gadig, 2000) - Brazilian large-eyed stingray, “raia	Asthma, pain relief caused in injuries by the species’ sting, burns	11,13,14

mariquita”		
Doradidae		
Franciscodoras marmoratus (Reinhardt, 1874) – “Urutu”	Injuries caused by itself	9
Lithodoras dorsalis (Valenciennes, 1840)	Swelling	11,13,14
Echeneidae		
Echeneis naucrates Linnaeus, 1758 - Live sharksucker, “rêmora”, “pegador”	Asthma, bronchitis	46, 50, 52
Electrophoridae		
Electrophorus electricus (Linnaeus, 1766) - Electric eel, “poraquê”	Sprains, bruises, bruises, insect bites, snake bite, asthma, flu, pain in general, muscle strain, rheumatism, osteoporosis, deafness, pneumonia, itching	11,12, 13,14, 15,46,48,61
Erythrinidae		
Erythrinus erythrinus (Bloch & Schneider, 1801) – “Matrôe”	Asthma	57
Hoplias malabaricus (Bloch, 1794) - Trahira, “traíra”	Ophthalmological problems, rheumatism, cataracts, wounds, snake bite, conjunctivitis, stroke, thrombosis, asthma, toothache, fever, earache, diarrhoea, deafness, boils, bleedings, alcoholism, tetanus, sore throat, itching, sprains, leucoma	11, 12, 13,14, 15, 42, 46, 48, 50, 52, 57,60,61
Hoplias lacerdae Miranda Ribeiro, 1908 - Giant trahira	Leucoma	58
Gadidae		
Gadus morhua Linnaeus, 1758[VU] – Atlantic cod, “bacalhau”	Boils	50
Ginglymostomatidae		
Ginglymostoma cirratum (Bonnaterre, 1788)[DD] - Nurse shark, “cação-lixa”	Rheumatism	11,13,14
Heptapteridae		
Pimelodella brasiliensis (Steindachner, 1876) – “mandim”	Injuries caused by that fish species	43
Rhamdia quelen (Quoy & Gaimard, 1824) – Mole, Catfish	Wekness	61
Megalopidae		

Megalops atlanticus (Valenciennes, 1847) - Tarpon, "camurupim", "cangurupim"	Stroke, headache, asthma, shortness of breath, thrombosis, chest pain, injuries caused by bang	11,13,14, 46, 52, 57
Muraenidae		
Gymnothorax funebris Ranzani, 1840 – Green moray, "moréia verde"	Bleeding (wounds)	11,13,14
Gymnothorax moringa (Cuvier, 1829) – Spotted moray, "moréia pintada"	Bleeding (wounds)	11,13,14
Gymnothorax vicinus (Castelnau, 1855) - Purplemouth moray, "moréia"	Bleeding (wounds)	11,13,14
Myliobatidae		
Aetobatus narinari (Euphrasen, 1790) – Spotted eagle ray, "raia- chita"	Asthma, pain relief caused in injuries by the species' sting, burns, haemorrhage	11,13,14
Narcinidae		
Narcine brasiliensis (Olfers, 1831) - Brazilian electric ray, "raia elétrica"	Toothache	46, 50, 52
Ogcocephalidae		
Ogcocephalus vespertilio (Linnaeus, 1758) - Batfish, "Peixe morcego"	Asthma, bronchitis	11,13,14
Pimelodidae		
Pseudoplatystoma corruscans (Spix & Agassiz, 1829) - Spotted sorubim, "surubim"	Flu	43
Prochilodontidae		
Prochilodus argenteus Spix & Agassiz, 1829 – "curimatá-pacú", "curimatá"	To avoid swelling of the breast feeding, mycosis	43
Serrasalmidae		
Serrasalmus brandtii (Lütken, 1875) - White piranha, "pirambeba"	Inflammations, sexual impotence	42, 57,61
Sciaenidae		
Cynoscion acoupa (Lacepède, 1801) - Acoupa weakfish, "pescada amarela"	Renal failure	11
Cynoscion leiarchus (Cuvier, 1830) - Smooth weakfish, "pescada branca"	Renal failure	11
Micropogonias furnieri (Desmarest, 1823) – Whitemouth croaker, "corvina"	Pain relief caused in injuries by the species' sting, cough, asthma, bronchitis	46, 50,52
Pachyurus francisci (Cuvier, 1830) - San	Asthma, urinary incontinence, backache	43

Francisco croaker, “cruvina-de-bico”		
Sparidae		
Calamus penna (Valenciennes, 1830) - Sheepshead porgy, “peixe-pena”	Asthma	46
Synbranchidae		
Synbranchus marmoratus Bloch, 1795 - Marbled swamp eel, “muçum”	Bronchitis	46, 52
Syngnathidae		
Hippocampus erectus Perry, 1810$^{VU/II/IN}$ – Horsefish, “cavalo-marinho”	Asthma	11
Hippocampus reidi (Ginsburg, 1933)$^{DD/II/IN}$ - Longsnout seahorse, “cavalo-marinho”	Asthma, edema, bronchitis, haemorrhage, haemorrhage in women, disorders after parturition (to accelerate recovery after parturition), gastritis, tuberculosis, to prevent abortion	11,12, 13,14,15, 46, 48, 50, 52, 60
Tetraodontidae		
Colomesus psittacus (Bloch & Schneider, 1801) - Banded puffer, “baiacu”	Breast cancer, backache, warts	11,13,14
Sphoeroides testudineus (Linnaeus, 1758) - Checkered puffer, “baiacu”	Rheumatism	12, 46, 48
Trichiuridae		
Trichiurus lepturus Linnaeus, 1758 - Largehead hairtail	Asthma	11
Urolophidae		
Urotrygon microphthalmum (Delsman, 1941) - Smalleyed round stingray, “raia”	Asthma, pain relief caused in injuries by the species’ sting, burns	11,13,14
AMPHIBIANS		
Bufonidae		
Rhinella schneideri (Werner, 1894) – Cururu toad, “sapo cururu”	Urinary incontinence, dental caries, cancer, wounds, boils, erysipelas acne, to induce abortion	46, 57
Rhinella jimi (Stevaux, 2002)	Gastritis, cancer	60
Leptodactylidae		
Leptodactylus cf. *labyrinthicus* (Spix, 1824) – South american pepper frog, “jia-de-peito”, “rã- pimenta”	Earache, rheumatism, joint pain, cancer, sore throat	46, 57
REPTILES		

Gekkonidae		
Hemidactylus mabouia (Moreau de Jonnes, 1818) - Afro-American house gecko, “lagartixa”, “briba”	Sore throat	11,13,14, 15
Iguanidae		
Iguana iguana (Linnaeus, 1758)[DD/II] - Common iguana, “camaleão”	Earache, erysipelas, asthma, rheumatism, edema, abscesses, joint pain, wounds, acne, athlete’s foot, sore throat, swelling, burn, tumour, to suck a splinter out of skin or flesh, boil, injuries caused by the spines of the ‘arraia’ and others fishes, inflammation, hernia	11,13,14,15, 46, 47, 55, 57, 60, 62
Teiidae		
Ameiva ameiva (Linnaeus, 1758) – Lizard, “sardão grande”	Inflammation, dermatitis, venereal diseases, snake bites	55, 57
Cnemidophorus gr. *ocellifer* (Spix, 1825) – Lizard, “sardão pequeno”	Inflammation, dermatitis, venereal diseases, snake bites	55, 57
Tupinambis merianae (Duméril & Bibron, 1839)[DD/II] – Lizard, “tegu”, “tejuaçú”	Earache, deafness, rheumatism, erysipelas, skin thorns and wounds, respiratory diseases, sore throat, snake bite, asthma, tumour, swelling, infection, bronchitis	11,13,14, 15, 46, 49, 59, 60, 61,62
Tupinambis teguixin (Linnaeus 1758)[DD/II] – Lizard, “tegu”, “tejuaçú”	Sexual impotence, rheumatism, erysipelas, dermatitis, snake bites, asthma, tetanus, earache, thrombosis, wounds, panaris, swelling, herpes zoster, irritation when milk teeth are erupting, jaundice, inflammation, tumour, sore throat, infection, bronchitis, injuries caused by the spines of the ‘arraia’, pain relief in injuries caused by snake bites, toothache, suck a splinter out of skin or fresh, headache, cough, stroke, coarse throat	11,13,14,15, 36, 37, 55
Tropiduridae		
Tropidurus hispidus (Spix, 1825) - Lizard, “lagartixa”, “catenga”	Alchoolism, dermatomycosis, warts, abscesses, boils, sore throat, erysipelas, healing of umbilical cord of newborn baby	11,13,14, 15, 55
Tropidurus semitaeniatus (Spix, 1825) – Lizard, “lagartixa-de-lajedo”	Measles, asthma, alcoholism, dermatomycosis, warts	46, 57

Tropidurus torquatus (Wied, 1820) – Lizard, “lagartixa”	Chicken pox	44,58
Uranoscodon superciliosus (Linnaeus, 1758), “tamaquaré”	As a sedative	11
Boidae		
Boa constrictor (Linnaeus, 1758)DD/II - Boa,“jibóia”	Rheumatism, lung disease, thrombosis, boils, tuberculosis, stomach ache, edema, snake bite, cancer, ache, swelling, to prevent abort, pain in the body, inflammation, athlete’s foot, calluses, tumours, cracks in the sole of the feets, goitre, sore throat, arthrosis, insect sting, dog bite, erysipelas, thrombosis, asthma, neck strain, strain muscle	11,13,14,15, 36,37, 39, 40, 46, 47, 49, 55, 57,61
Corallus caninus (Linnaeus, 1758)DD/II - American emerald tree boa, “cobra papagaio”	Pain relief caused by sting of animals	39, 40
Corallus hortolanus (Linnaeus, 1758)DD/II – Snake	To assist in removing spines or other sharp structures from the skin , rheumatism	11
Eunectes murinus (Linnaeus, 1758)DD/II - Anaconda, “sucurujú”, “sucuri”	Wounds, skin problems, bruises, sprains, arthrosis, rheumatism, boils, sexual impotence, headache, sore throat, thrombosis, swelling, tumour, asthma, muscle strain, numbness, syphilis, to reduce pain, luxation	11,13,14,15, 39, 46, 52,58,61
Epicrates cenchria (Linnaeus, 1758)DD/II – Brazilian rainbow boa, “salamanta”	Rheumatism, pain in articulations, injuries caused by itself, sore throat	11,13,14, 15, 57, 36, 37, 39, 40, 46, 49, 55,61
Colubridae		
Leptophis ahetula (Linnaeus, 1758) - Parrot snake, “cobra cipó”	Pain relief caused by sting of animals	11, 15, 39, 40
Mastigodryas bifossatus (Raddi, 1820) – Rio tropical racer, “jaracuçú”	Snake bites	55
Oxyrhopus trigeminus Duméril, Bibron & Duméril, 1854 – “Coral falsa”	Rheumatism	11, 15, 40, 55
Spilotes pullatus (Linnaeus, 1758) - Tiger snake	Pain relief in injuries caused by sting of insects and snake bite	11,13
Crotalidae		
Crotalus durissus (Linnaeus, 1758)DD/III -	Asthma, snake bite, thrombosis, wounds,	10, 11, 12, 13,14, 15, 36, 37, 39, 40,48, 49, 44, 46,

Neotropical rattlesnake, "cascavel"	luxation, rheumatism, pain in the legs, erysipelas, deafness, epilepsy, skin diseases, tuberculosis, hanseniasis, backache, tumour, boil, headache, earache, osteoporosis, sore throat, toothache, pain relief in injuries caused by sting of insects and snake bite, irritation when milk teeth are erupting,	55, 58, 59, 60, 62
Elapidae		
Micrurus ibiboboca (Merrem, 1820) - "Cobra-coral"	Rheumatism, snake bite	55
Viperidae		
Bothrops leucurus Wagler, 1824 - Lance head, "jararaca"	Tumour, boils	39
Lachesis muta (Linnaeus, 1766) - Bushmaster,"surucucu pico-de-jaca"	Rheumatism, swelling, tumour, boil, pain relief in injuries caused by sting of insects and snake bite	11,13,14,15, 39,40, 55
Chelidae		
Phrynops geoffroanus (Schweigger, 1812) - Geoffroy's side-necked turtle, "cágado"	Asthma, sore throat, swelling, earache, rheumatism, arthrosis, healing of umbilical cord of newborn baby, mumps	11,13,14, 15, 58, 60,62
Mesoclemmys tuberculata (Luederwaldt, 1926) – Tuberculate toadhead turtle, "cágado", "cágado-d'água"	Rheumatism, discharge, thrombosis, bronchitis, diarrhoea, haemorrhag, asthma, sore throat, hoarseness	36, 37, 55, 57
Cheloniidae		
Caretta caretta (Linnaeus, 1758)[VU/I/IB] - Loggerhead turtle, "tartaruga cabeçuda"	Injuries caused by bang, toothache, diabetes, headache, backache, wounds, cough, bronchitis, asthma, thrombosis, rheumatism, stroke, hoarseness, flu, backache, earache, sore throat, swelling	11,13,14, 38, 46, 50, 52
Chelonia mydas (Linnaeus, 1758)[VU/I/IB] - Green sea turtle, "tartaruga verde", "aruanã"	Injuries caused by bang, toothache, diabetes, headache, backache, wounds, cough, bronchitis, asthma, flu, thrombosis, rheumatism, toothache, stroke, hoarseness, earache, sore throat, swelling, whooping cough, arthritis, erysipelas, boil, wounds, arthrosis, inflammation	11,13,14, 38,46, 50, 52
Eretmochelys imbricata (Linnaeus,	Injuries caused by bang, toothache, diabetes,	11,13,14, 38,46, 50, 52

1766)[EN/I] - Atlantic hawksbill, "tartaruga de pente"	headache, backache, wounds, cough, bronchitis, asthma, thrombosis, stroke, hoarseness, flu, rheumatism, earache, sore throat, swelling	
Lepidochelys olivacea (Eschscholtz, 1829)[EM/I/IB]	Injuries caused by bang, toothache, diabetes, headache, backache, wounds, cough, flu, bronchitis, asthma, thrombosis, rheumatism, stroke, hoarseness	46, 50, 52
Dermochelyidae		
Dermochelys coriacea (Vandelli, 1761)[CR/I/IB] - Leatherback turtle, "tartaruga de couro"	Rheumatism, earache, sore throat, swelling	11,13,14, 38
Geoemydidae		
Rhinoclemmys punctularia (Daudin, 1802) - Spot-legged turtle	Wounds, tumour, erysipelas, earache, rheumatism	11,13,14
Testudinidae		
Chelonoidis carbonaria (Spix, 1824)[DD/II] - Red-footed tortoise, "jabuti"	Catarrh, erysipelas, bronchitis, to stop the sensation to getting thirsty, asthma	11,12, 13,14, 15, 44, 46, 48, 61,62
Chelonoidis denticulata (Linnaeus, 1766)[VU/II] – Yellow-footed tortoise, "jabuti"	Sore throat, rheumatism, hernia, wounds, leishmaniosis, varicocele, earache	11,13,14,15,60
Alligatoridae		
Caiman crocodilus (Linnaeus, 1758) [II] - Common cayman, "jacaré tinga"	Asthma, stroke, bronchitis, backache, earache, rheumatism, thrombosis, sexual impotence, snake bites (antidote), evil eye, irritation when milk teeth are erupting, discharge, swelling, scratch, athlete's foot, ophthalmological problems, asthma, sore throat, amulet used as a protection against snake bite, rheumatism, hernia, prostate problems, infection, thrombosis	11,12, 13,14, 37, 41, 46, 47, 48, 52, 55, 57
Caiman latirostris (Daudin, 1801) [II] – Cayman, "jacaré-do-papo-amarelo"	Asthma, sore throat, amulet used as a protection against snake bite, rheumatism, irritation when milk teeth are erupting, hernia, prostate problems	11,13,14,15, 37, 49,58,60,61
Melanosuchus niger (Spix, 1825) [II] - Black cayman, "jacare açú"	Thrombosis, infection, swelling, asthma, amulet used as a protection against snake bite, injuries caused by spines of the 'arraia', pain relief in injuries caused by snake bites	11,13,14

Paleosuchus palpebrosus (Cuvier, 1807) [II] - Cayman, "jacaré coroa", "jacaré", "jacaré-preto", "crocodilo"	Snake bite, asthma, stroke, rheumatism, thrombosis, backache, sexual impotence, edema, mycosis, evil eye, irritation when milk teeth are erupting, snake bite (antidote), discharge, sore throat, amulet used as a protection against snake bite, hernia, prostate problems	11,13,14, 15, 46, 49, 52,58
BIRDS		
Anatidae		
Anser anser (Linnaeus, 1758). – Greylag goose, "ganso"	Laryngitis, pharyngitis, tonsillitis	57, 60
Anhimidae		
Anhima cornuta (Linnaeus, 1766) – Horned screamer, "anuhma"	Intoxication from poisonous animals	9
Ardeidae		
Ardea cocoi (Linnaeus, 1766) – White-necked Heron	Swelling, inflammation, injuries caused by the spines of the 'arraia' and others fishes, asthma, boil, tumour, inflammation, rheumatism, earache	11, 13
Ardea alba Linnaeus, 1758 – Galça	Bronchitis, pneumony	58
Tigrisoma lineatum (Boddaert, 1783) – socó, Rufescent Tiger-Heron	Bronchitis,pneumony	58
Psittacidae		
Amazona aestiva (Linnaeus, 1758)	Asthma	
Cathartidae		
Coragyps atratus (Bechstein, 1793) - Black vulture, "urubu", "urubu-preto"	Deafness, bronchitis, anaemia, alcoholism, asthma, flu (catarrh), earache	11,13,14, 15, 57, 58,60,61,62
Cracidae		
Penelope jacucaca (Spix, 1825)[VU/IB] – White-browed guan, "jacu"	Insomnia	46
Penelope superciliaris Temminck, 1815 - Rusty-margined Guan	Asthma	61
Ciconidae		
Ciconia maguari (Gmelin, 1789) - Maguari stork	Injuries caused by the spines of the 'arraia' and others fishes, thrombosis	11, 13
Columbidae		

Leptotila rufaxilla (Richard & Bernard, 1792) – Gray-fronted dove, 'juriti"	Thrombosis	46
Columba livia (Gmelin, 1789) – Rock pigeon, "pombo"	Asthma, laryngitis, pharyngitis, tonsilite	57
Corvidae		
Cyanocorax cyanopogon (Wied, 1821) – White-naped jay, "can-can"	Asthma	46,58, 60,62
Cuculidae		
Crotophaga ani Linnaeus, 1758 – Smooth-billed ani	Bronchitis, thrombosis, asthma, whooping cough	11,13, 15, 57,58
Guira guira (Gmelin, 1788) – Guira cuckoo, "anum branco"	Asthma	57
Charadriidae		
Vanellus chilensis (Molina, 1782) – Southern lapwing, "quero-quero"	To stay awake	44, 46
Emberezidae		
Coereba flaveola (Linnaeus, 1758) – Bananaquit, "caga-sebo"	Thrombosis	46
Furnaridae		
Furnarius rufus (Gmelin, 1788)- Rufous hornero, "maria-barreira"	Mumps	52
Meleagrididae		
Meleagris gallopavo Linnaeus, 1758 – turkey, "peru"	Asthma	11,13,14, 59,62
Phasianidae		
Gallus gallus (Linnaeus, 1758) - Domestic chicken, "galinha"	Catarrh, fever, warts, haemorrhage, bronchitis, nasal congestion, flu, skin thorns and wounds, asthma, sore throat, tumour, poor digestion, healing of umbilical cord of newborn baby, swelling, cough, tuberculosis, earache, tonsillitis, rheumatism, diarrhea	11,12, 13,14,15, 37, 37, 44, 46, 47, 48, 49, 52, 57, 58, 59, 60,61,62
Pavo cristatus Linnaeus, 1758 - Indian peafowl	Thrombosis, epilepsy	42,60
Rallidae		
Aramides cajanea (Statius Muller, 1776) – Grey-necked wood-rail, "saracura"	Evil eye	46
Numididae		

Numida meleagris Linnaeus, 1758 - Helmeted Guineafowl, "Guiné"	Pertussis	60
Struthionidae		
Struthio camelus Linnaeus, 1766 - Common Ostrich, "Avestruz"	Osteoporosis	60
Rheidae		
Rhea americana (Linnaeus, 1758) [II] – Greater rhea, "ema"	General aches, rheumatism, thrombosis, strokes	11, 12, 15, 44, 46,48
Tinamidae		
Crypturellus noctivagus (Wied, 1820)[VU/IB] - Yellow-legged tinamou, "zabele"	Thrombosis, stroke	44, 46,58,61
Nothura boraquira (Spix, 1825) – White-bellied nothura, 'codorna"	Thrombosis, stroke	44, 46,59,61
Nothura maculosa (Temminck, 1815) - Spotted Nothura English, Codorniz	Snake bite	60,62
Rhynchotus rufescens (Temminck, 1815) – Red-winged tinamou, "perdiz"	Snake bite, thrombosis, snake bites (antidote),	46, 49,61
Trochilidae		
Eupetomena macroura (Gmelin, 1788) – Swallow-tailed hummingbird, "beijola", "beija-flor"	Cardiopathies, asthma, flu, ache	57
Tyrannidae		
Fluvicola nengeta (Linnaeus, 1766) - Masked water-tyrant, "lavandeira"	Boils	57
Pitangus sulphuratus (Linnaeus, 1766) - Great Kiskadee, bem-te-vi	Earache	61
Cotingidae		
Procnias nudicollis (Vieillot, 1817) , araponga, bare-throated Bellbird	Tuberculosis	58
MAMMALS		
Agoutidae		
Agouti paca (Linnaeus, 1766) [III] – Spotted paca, "paca"	Wound in the breast caused by suckling, ophthalmological problems, stomach disorders, pterygium, to suck a splinter out of skin or flesh, injuries caused by the spines of	11,13,14, 15, 46, 57,61

	'arraia', control cholesterol level	
Balaenopteridae		
Balaenoptera acutorostrata Lacépède, 1804 [I] – Minke whale, "baleia minke"	Rheumatism, sore throat, wounds	11,13,14, 15
Bovidae		
Bos taurus Linnaeus, 1758 – Cow, "vaca"	Thrombosis, evil eye, amulet used as a protection against snake bite, baldness, sexual impotence, measles, varicella, anaemia, whooping cough, alcoholism, rheumatism, inflammation, asthma, cough, sore throat, wounds, cracks in the sole of the feet, dizziness	10, 11,13,14,15, 44, 46, 47, 57, 58, 59, 60,61,62
Bubalus bubalis (Linnaeus, 1758) – Water buffalo (feral), "búfalo"	Rheumatism, osteoporosis, thrombosis	11,15
Ovis aries (Linnaeus, 1758) – Sheep, "carneiro"	Edema, fractures, erysipelas, herpes zoster, backache, swelling, to assist children who take longer than usual to start walking, arthritis, arthrosis, rheumatism, muscle strain, inflammation, luxation, cracks in the sole of the feet, joint pain	10, 11,13,14, 15, 44, 46, 47 57, 59, 60,61,62
Capra hircus Linnaeus, 1758 – Domestic goat, "bode"	Evil eye, snake bite, muscle strain	46 ,46,59
Bradypodidae		
Bradypus variegatus Shinz, 1825 – Brown-throated three-toed sloth, "Preguiça pequena"	Thrombosis	11,15,57
Canidae		
Canis lupus (Linnaeus, 1758) – Domestic dog, "cachorro"	Chicken pox, mumps, smallpox, asthma, varicella, measles, menstrual cramps	11,13,14, 44, 46, 57,58,61
Cerdocyon thous (Linnaeus, 1766) [II] – Crab-eating fox, "raposa"	Rheumatism, flu, haemorrhoids, disorders after parturition (to accelerate recovery after parturition)	11,13,14, 15, 59, 60,61,62
Chrysocyon brachyurus (Illiger, 1815) [II/IB] – Maned wolf, "lobo-guará"	Epilepsy	36, 37, 46
Dusicyon thous – Linnaeus, 1766 – Crab-eating fox, "raposa"	Alcoholism, thrombosis, rheumatism, ophthalmological problems, diabetes, urinary infection	57

Caviidae		
Cavia aperea Erxleben, 1777 – "Preá"	Inflammation	57, 62
Kerodon rupestris (Wied-Neuwied, 1820) – "Mocó"	Constipation	46,60
Cebidae		
Alouatta belzebul (Linnaeus, 1766)[CR/IB] – Red-handed howler monkey, "guariba", "macaco"	Whooping cough, sore throat, asthma	11,13
Cebus apella (Linnaeus, 1758) [II] – Brow capuchin, "capuchin", "macaco", "macaco-prego"	Insect sting	11,13,14,15
Cervidae		
Blastocerus dichotomus (Illiger, 1815)[VU/I/IB] – Marsh deer, "cervo-do-pantanal"	Diarrhoea, vomit	9
Mazama americana (Erxleben, 1777)[DD/III] – Red brocket, "veado gaedo"	Stroke	44
Mazama cf. *gouazoupira* (G. Fischer, 1814) – Gray brocket, "veado-catingueiro"	Asthma, edema, rheumatism, snake bite, thrombosis, to assit children who take longer than usual to start walking, tootache, wounds, sprains	12, 46, 48, 57
Dasypodidae		
Dasypus novemcinctus (Linnaeus, 1758) – Nine-banded armadillo, "tatu galinha"	Thrombosis, insects bite, scorpions bite, edema, asthma, deafness, earache, evil eye	11,13,14,15, 47, 57
Euphractus sexcinctus (Linnaeus, 1758) – Six-banded armadillo "tatu peba"	Wounds, earache, evil eye, asthma, sore throat, pneumonia, sinusitis, deafness, coarse throat	11,13,14, 15,46, 47,59,60
Tolypeutes tricinctus (Linnaeus, 1758)[VU/IB] – Brazilian three-banded armadillo, "tatu-bola"	Thrombosis, rheumatism	46,47
Dasyproctidae		
Dasyprocta prymnolopha Wagler, 1831 – Black-rumped agouti, "Cutia"	Asthma, thrombosis	57
Delphinidae		
Sotalia fluviatilis Gervais & Deville, 1853)[DD/I] – Gray dolphin, gray river dolphin, "boto"	Asthma, headache, rheumatism, hernia, womb disorders, sore throat, injuries caused by the spines of the 'arraia', swelling, haemorrhoids inflammation, wounds,	11,13,15

	earache, erysipelas, athlete´s foot, tumour, cancer	
Sotalia guianensis (P. J. Van Bénéden, 1864) – Guianan river dolphin, "boto"	Asthma, headache, rheumatism, hernia, womb disorders, sore throat, injuries caused by the spines of the 'arraia', swelling, haemorrhoids inflammation, wounds, earache, erysipelas, athlete´s foot, tumour, cancer	11,13,15
Didelphidae		
Didelphis albiventris (Lund, 1840) – Common opossum, "timbú"	Boils	11,13,14,58,61
Didelphis marsupialis (Linnaeus, 1758) – Southern opossum, "mucura", "gambá", "saruê"	Acne, wounds, bronchitis, joint pain, stomach ache, rheumatism, diarrhoea, inflammation, erysipelas, pain in gestation, asthma, headache, toothache, earache, sore throat	11,13,14,15
Erethizontidae		
Coendou prehensilis (Linnaeus, 1758) – Brazilian porcupine, "coandú", "porco espinho"	Bronchitis, thrombosis, epilepsy, stroke, abscesses, conjunctivitis, asthma	10, 11,12, 13,14, 15, 36, 37, 44, 48, 49, 57,60,61
Equidae		
Equus asinus Linnaeus, 1758 – Asino, ass, "jumento"	Snake bite (antidote), whooping cough, asthma	11,13,14, 15, 44, 46,60
Equus caballus (Linnaeus, 1758) – Horse, "cavalo"	Cough, deep cuts; dermatosis, wounds	46,47
Felidae		
Felis silvestris Schreber, 1775 – Domestic cat, "gato"	Asthma	46, 47,61
Hydrochaeridae		
Hydrochaeris hydrochaeris (Linnaeus, 1766) – Capybara, "capibara", "capivara"	Thrombosis, conjunctivitis, venereal disease, rheumatism, earache, strengthen bones, liver pain, bronchitis, asthma, wounds, erysipelas, cough	11,13, 15, 36, 37 , 46, 49,57, 58,61
Iniidae		
Inia geoffrensis (Blainville, 1817)[VU/II] – Amazon river dolphin, "boto rosa"	Asthma, headache, rheumatism, hernia, womb disorders, sore throat, injuries caused by the spines of the 'arraia', swelling, haemorrhoids inflammation, wounds,	11,13

	earache, erysipelas, athlete’s foot, tumour, cancer	
Leporidae		
Sylvilagus brasiliensis (Linnaeus, 1758) – Forest rabbit, tapeti, “coelho”, “coelho-do-mato”	Thrombosis, conjunctivitis, boils, burns	11,13,14, 46, 57,61
Mustelidae		
Conepatus semistriatus (Boddaert, 1785) – Striped hog-nosed skunk, “cangambá”, “gambambá”, tacaca	Rheumatism	46,47, 60,62
Conepatus chinga (Molina, 1782) – Gambá, Molina's Hog-nosed Skunk	Thrombosis, Rheumatism	61
Lontra longicaudis (Olfers, 1818)[DD/I] – “Lontra”, Neotropical Otter	Thrombosis	46
Myrmecophagidae		
Myrmecophaga tridactyla Linnaeus, 1758[VU/II/IB] – Giant anteater, “tamanduá-bandeira”	Thrombosis, stroke	44, 46, 47
Myrmecophaga tetradactyla (Linnaeus, 1758) – Collared anteater, “tamanduá”	Edema, thrombosis	11,13,14, 57, 61,62
Procyonidae		
Nasua nasua (Linnaeus, 1766)[/III] – South American coati, “coati”, “quati”	Sexual impotence, wounds, skin burns, snake bites, backache	11,12 ,13,14, 36, 37, 46, 49, 48
Procyon cancrivorus (G. [Baron] Cuvier, 1798) – Crab-eating raccoon, “guaxinim”	Rheumatism, epilepsy, thrombosis, snake bite	11,13,14, 15, 49, 57,61
Physeteridae		
Physeter catodon Linnaeus, 1758[VU/I/II/IB] – Sperm whale, cachelot, “cachalote”	Asthma, backache, rheumatism, sore throat, wounds	11,13,14, 15,50
Suidae		
Sus scrofa (Linnaeus, 1758) – Wild boar, “porco”	Acne, boils, tumours, asthma, Athlete’s foot, berne, wounds	11,13,14, 44, 46, 47, 44,59,61
Tapiridae		
Tapirus terrestris (Linnaeus, 1758)[VU/II] – Brazilian tapir, “anta”	Rheumatism, arthrosis, osteoporosis, bursitis, muscular pain, asthma, tonsillitis	11,15
Tayassuidae		
Pecari tajacu Linnaeus 1758 [II/III] – Collared peccary, “porco-do-mato”, “caititu”	Thrombosis, bronchitis, stroke	11,15, 44, 46,61

Tayassu pecari (Link, 1795) [II] – White-lipped peccary "porco-do-mato", "queixada"	Thrombosis, stroke	44, 46
Trichechidae		
Trichechus inunguis (Natterer, 1883)[VU/I/IB] – Amazonian manatee, "peixe-boi"	Sprains, vaginal discharge, injuries caused by bang, burns, asthma, menstrual cramps, rheumatism, sore throat, wounds, muscle strain, suck a splinter out of skin or fresh, tumour, backache, hernia, arthrosis, luxation, menstrual cramps, insects bite	11,12, 13,14,15, 46,48
Trichechus manatus (Linnaeus, 1758)[CR/I/IB] – Manatee, "peixe-boi"	Sprains, vaginal discharge, injuries caused by bang, burns, asthma, menstrual cramps, rheumatism, sore throat, wounds, muscle strain, suck a splinter out of skin or fresh, tumour, backache hernia, arthrosis, luxation, menstrual cramps, insects bite	11,12, 13,14,15, 46,48

Categories of IUCN Red List: CR – Critically andangered, EN – Endangered, VU – Vunerable, – Lower risk, DD – Deficient data and NE - Note Evaluated
CITES Appendix (I, II and III)
IN – Anexo 2 – Instrução Normativa n. 5/2004/MMA
IB - Brazil's official list of endangered species
